# Editorial: Immunological Memory to Fungal Infections and Vaccine Development

**DOI:** 10.3389/fimmu.2022.880037

**Published:** 2022-04-27

**Authors:** André Moraes Nicola, Jigar V. Desai, Marc Swidergall, Muki Shey, Ivy M. Dambuza

**Affiliations:** ^1^ Faculty of Medicine, University of Brasília, Brasília, Brazil; ^2^ Fungal Pathogenesis Section, Laboratory of Clinical Immunology and Microbiology, National Institute of Allergy and Infectious Diseases, National Institutes of Health (NIH), Bethesda, MD, United States; ^3^ Department of Medicine, David Geffen School of Medicine at University of California, Los Angeles, Los Angeles, CA, United States; ^4^ Department of Medicine & Wellcome Centre for Infectious Disease Research in Africa (CIDRI-Africa), Institute of Infectious Disease and Molecular Medicine, Faculty of Health Sciences, University of Cape Town, Cape Town, South Africa; ^5^ MRC Centre for Medical Mycology, University of Exeter, Exeter, United Kingdom

**Keywords:** vaccine, immunological memory, fungi, aspergillosis, paracoccidioidomycosis, histoplasmosis, mycoses, candidiasis

Throughout human history, infectious diseases have been one of the leading causes of death. However, due to advances in modern medicine, life expectancy and the total population increased considerably. Among the key drivers of this major change, vaccines have led to the elimination or significant reduction in the burden of several viral and bacterial diseases. Unfortunately, none of the clinically available vaccines target fungal diseases, which have been estimated to cause some 1.5 million deaths each year (1). Given that the more traditional vaccine technologies failed to yield effective immunization for invasive mycoses, a deeper understanding of the host response to fungi and the development of new technologies are both necessary before vaccine-mediated reductions in the burden of systemic fungal diseases are apparent. This Research Topic highlights some of the cutting-edge research and technological development done recently in this area.

While some invasive fungal infections are acquired, others originate by commensal outgrowth. Given that vaccines work by inducing immunological memory, it is imperative to understand adaptive responses in this context. In a review, LeibundGut-Landmann describes what is presently known about the role of a specific immune cell type in this process: tissue-resident memory T cells. Strategically located in peripheral tissues and especially in the mucosae, these cells trigger fast and effective responses against antigens with which the host has had previous contact. Thus, the presence of these cells in immunized individuals augments the immediate immune response beyond what is afforded by preformed innate immune effectors and could play strategic roles in antifungal immunity at barrier sites, in particular the gut, skin or respiratory mucosa. However, the biggest challenge is to devise strategies and technologies that efficiently result in memory T cells establishing residency on specific tissues after immunization, even more so when we consider the tendency of mucosal immune responses towards tolerance.

Other challenges that have so far precluded successful vaccines in medical mycology have been highlighted by Biswas in his review. Some of them are related to characteristics of these microorganisms and their interactions with the human host, such as the fact that *Candida* spp. are commensal microbes against which protective immunity is consequently very hard to achieve. Other difficulties stem from the fact that a large proportion of patients are immunocompromised due to AIDS or one of the many therapeutic tools used in modern medicine such as stem cell transplantation, cancer chemotherapy, post-transplantation immunosuppression and broad-spectrum antimicrobials. Antifungal vaccines have also been hindered by the fact that some of these illnesses are neglected tropical diseases and that most of them tend to affect specific groups of people instead of entire populations, making their development less economically interesting for the pharmaceutical industry. Despite all these difficulties, Biswas underscores several interesting *in vitro* and *in vivo* pre-clinical studies and even an encouraging phase 1 clinical trial (2) of vaccine candidates, some of them against specific fungal pathogens and others against mycoses in general.

These pan-fungal vaccines are a possibility that stems from another study in this Research Topic (Kischkel et al.). Kischkel et al. used two different but complementary strategies to seek novel *Histoplasma capsulatum* protein epitopes that elicit robust immune responses in mice. One strategy revealed proteins that were recognized by antibodies, whereas the other showed peptides that were presented by dendritic cells to activate T cells. After identifying such immunogenic epitopes, they focused on those present in proteins that are highly conserved among different pathogenic fungal species, which could lead to cross-species immunogenicity. When these peptides were tested, they led to T cell activation and secretion of Th1- and Th17-type cytokines, a promising indication that they might enhance the kind of immune response that is protective in most systemic and mucosal mycoses.

Another genus of dimorphic fungi, *Paracoccidioides*, was the subject of a paper in this Research Topic (Silva et al.). Studies with *P. brasiliensis* have previously shown that peptides derived from the immunodominant gp43 protein can be protective in animal models (3). However, gp43 is poorly conserved in other *Paracoccidioides* species, so Silva et al. used an immunoproteomic approach to discover new candidate vaccine antigens. They were able to find immunogenic peptides that resulted in effective activation of primed T cells, a first indication that they might prove useful in future vaccines.

In contrast with the approach used in the previous two manuscripts, which sought new vaccine antigens, Rayens et al. tested whether the *Aspergillus fumigatus* homolog of a protein they had previously found to be highly immunogenic in *Pneumocystis jirovecii* was also protective in experimental aspergillosis. Mice that were immunized with the *A. fumigatus* KEX1 protein had lower lung fungal burden, less severe pathology and increased survival after infection with *A. fumigatus* when compared with controls. Interestingly, this protection was observed even though the mice were immunosuppressed with corticosteroids or tacrolimus after the vaccination and prior to infection, a result that is very significant when considering that invasive aspergillosis happens almost exclusively in people whose immune responses are hampered.

The papers that are part of this Research Topic point to much of what we still do not know about the immunological memory to fungi, a gap that must be bridged before we can develop vaccines that successfully protect against severe fungal diseases. On the other hand, the new perspectives, and exciting experimental results presented here offer much hope towards decreasing the public health burden of the invasive mycoses.

## Author Contributions

All authors listed have made a substantial, direct, and intellectual contribution to the work and approved it for publication.

## Funding

AMN was funded by the Fundação de Apoio à Pesquisa do Distrito Federal (FAP-DF) awards 0193.001048/2015 and 0193.001561/2017 and the Conselho Nacional de Desenvolvimento Científico e Tecnológico (CNPq) grant 437484/2018-1. JVD was supported by the Division of Intramural Research (DIR) of the National Institute of Allergy and Infectious Diseases (NIAID). MaS was supported by NIH grant R00DE026856, an American Association of Immunologists Careers in Immunology Fellowship, and a TLI seed grant. MuS was supported by the Wellcome Trust (grant#: 211360/Z/18/Z) and the National Research Foundation of South Africa (NRF, grant#: UID127558). IMD was supported by the Wellcome Trust Strategic Award in Medical Mycology and Fungal Immunology (grant# 097377).

## Conflict of Interest

The authors declare that the research was conducted in the absence of any commercial or financial relationships that could be construed as a potential conflict of interest.

## Publisher’s Note

All claims expressed in this article are solely those of the authors and do not necessarily represent those of their affiliated organizations, or those of the publisher, the editors and the reviewers. Any product that may be evaluated in this article, or claim that may be made by its manufacturer, is not guaranteed or endorsed by the publisher.

## References

[1] Brown GD Denning DW Gow NA Levitz SM Netea MG White TC. Hidden Killers: Human Fungal Infections. Sci Transl Med (2012) 4(165):165rv113. doi: 10.1126/scitranslmed.3004404 23253612

[2] Schmidt CS White CJ Ibrahim AS Filler SG Fu Y Yeaman MR. NDV-3, a Recombinant Alum-Adjuvanted Vaccine for *Candida* and *Staphylococcus aureus*, Is Safe and Immunogenic in Healthy Adults. Vaccine (2012) 30(52):7594–600. doi: 10.1016/j.vaccine.2012.10.038 PMC3513491 23099329

[3] Taborda CP Juliano MA Puccia R Franco M Travassos LR. Mapping of the T-Cell Epitope in the Major 43-Kilodalton Glycoprotein of *Paracoccidioides Brasiliensis* Which Induces a Th-1 Response Protective Against Fungal Infection in BALB/c Mice. Infect Immun (1998) 66(2):786–93. doi: 10.1128/IAI.66.2.786-793.1998 PMC107971 9453642

